# Role of Botulinum Toxin Type-A (BTX-A) in the Management of Trigeminal Neuralgia

**DOI:** 10.1155/2013/831094

**Published:** 2013-10-01

**Authors:** Gaurav Verma

**Affiliations:** Himachal Institute of Dental Sciences, Paonta Sahib, Himachal Pradesh, House No. 521-A, Model Town, Yamuna Nagar, Haryana 135001, India

## Abstract

Trigeminal neuralgia (TN) is a clinical condition characterized by paroxysmal attacks of severe and electric shock-like pain along the distribution of one or more branches of the trigeminal nerve. Various medicinal or surgical modalities have been employed in the past with variable success. Newer methods were tried in search of permanent cure or long-lasting pain relief. The purpose of this paper is to present the review of the literature regarding the use of botulinum toxin type-A (BTX-A) in the management of trigeminal neuralgia.

## 1. Introduction

The English philosopher John Locke in 1677 firstly described the trigeminal neuralgia (TN). In 1756, Nicolaus Andre coined the term “tic douloureux” for the condition characterized by trigeminal neuralgia pain as well as muscle spasm. In 1773, John Fothergill gave detailed description of the trigeminal neuralgia [1].

Trigeminal neuralgia (TN) is a painful disorder of the trigeminal nerve characterized by paroxysmal attacks of severe, electric shock-like pain typically present on one side of the face. Pain is unilateral and follows one or more of the distributions of the trigeminal nerve. Mandibular and maxillary divisions are more commonly involved than ophthalmic division. There is a slight predilection for female sex. The right side of the face is more commonly involved than the left side of the face. Trigeminal neuralgia affects approximately 1 person in 25,000 people. It is more prevalent in middle or old age group people [1–3]. 

Various treatment modalities for trigeminal neuralgia include medicinal management, peripheral nerve injection of local anesthetic or alcohol, peripheral neurectomies, alcohol injection of trigeminal ganglion, and intracranial neurosurgical procedures [4]. 

## 2. Botulinum Toxin (BTX)

The botulinum toxin (BTX) is a naturally occurring neurotoxin that is produced by gram-positive anaerobic bacteria *Clostridium botulinum*. There are seven distinct antigenic subtypes of botulinum toxin. Botulinum toxin type-A (BTX-A) is the most commonly used form for cosmetic purposes [5]. 

The BTX-A is prepared by Hall strain *Clostridium botulinum* fermentation. It is precipitated, filtered, and further processed into a vacuum-dried fine powder. A standard vial of BTX-A contains 100 units of toxin, 0.5 mg of human albumin, and 0.9 mg of sodium chloride [6]. 

## 3. Risks Factors and Adverse Effects of Botulinum Toxin (BTX) 

Botulinum toxin has a high safety profile, reflected by paucity of documented irreversible medical complications [7, 8]. Botulinum toxin is most effective at local injection site. Minute quantity of toxin may spread to adjacent tissues or enter the circulatory system. Due to this diffusion, it can produce regional or systemic side effects [9]. Due to diffusion of toxin into systemic circulation, there is a possibility of antibody production and a potential for immune-mediated and other long-term complications. Development of antibodies to toxin may be related to exposure to high doses [9]. The main clinical consequence of these antibodies is decreased efficacy of botulinum toxin. The possibility of long-term complications, immune-mediated damage, and idiosyncratic reactions is unknown. After 1997, the botulinum toxin formulations contain lower concentration of human albumin which may lead to decreased clinical antigenicity [6, 10]. 

## 4. Contraindications for Use of Botulinum Toxin (BTX)

Botulinum toxin is contraindicated in patients with known allergy or hypersensitivity reaction to botulinum toxin or human albumin. It is not recommended in individuals with neurological diseases such as multiple sclerosis, myasthenia gravis, and Eaton-Lambert syndrome. Botulinum toxin injections must be avoided or delayed in patients taking aminoglycosides as they interfere with neuromuscular transmission [11]. 

The use of botulinum toxin during lactation and pregnancy remains controversial. There are few documentations of the safe use of botulinum toxin in pregnancy without any complications [12]. FDA classified it under category C drug, indicating that its safety profile in pregnancy has not been studied. Majority of clinicians recommended avoiding its use until the end of pregnancy. Treatment in nursing mothers is also controversial. Although botulinum toxin effects seem to be localized, its concentration in human milk and its effect on nursing baby are not known. Until safe guidelines are available for its use, it should be better avoided in pregnant and lactating mothers [13]. 

## 5. Mechanism of Action of Botulinum Toxin (BTX)

The main effect of botulinum toxin type-A (BTX-A) is on muscle contraction because of its binding to the presynaptic nerve terminals, thus inhibiting the release of the acetylcholine (Ach). It produces its antinociceptive effect in trigeminal neuralgia by several mechanisms. A direct analgesic action has been recognized, suggesting that BTX may act through an alternative mode of action [14]. 

Most hypotheses assume that BTX-A inhibits the release of Ach as well as other neurotransmitters. Inhibition of the release of these neurotransmitters from nociceptive nerve endings can lead to pain relief. Second site for BTX-A action to produce analgesic effect could be postganglionic sympathetic nerve endings utilizing norepinephrine (NE) and adenosine triphosphate (ATP) as neurotransmitters. NE is increased in chronic pain, and ATP is involved in the stimulation of muscle nociceptors. It has been postulated that BTX-A may inhibit the release of these neurotransmitters and produce analgesic effect in cases of sympathetically maintained pain involved in complex regional pain syndrome [15]. 

One fundamental feature of orofacial pain is its degree of complexity. Inflammation of the nervous system may be involved in the alteration of the peripheral as well as central component of sensory nervous system. This alteration of the sensory nervous system involves various mediators, receptors, conduction, transmission, phosphorylation process, and so forth [16–18]. Decreased sensitization of peripheral sensory nerve by stimuli may contribute to less alteration and responsiveness of peripheral and/or central component of sensory nervous system [17]. BTX-A can be considered as a safe, regional, and long-acting agent that alters the chemical environment around the peripheral nerve involved in the neurogenic inflammation [19]. 

Local inflammation leads to peripheral sensitization of nociceptive neurons and thus increased pain inputs. This will lead to increased release of substance P (SP) in the spinal cord leading to central sensitization [20]. Substance P (SP) is a peptide released both centrally and peripherally by nociceptive primary afferent C-fibers. BTX-A inhibits the release of SP, thus producing the analgesic effect seen in primary headache disorders [21]. 

Release of glutamate peripherally results in inflammation, pain, and edema [22]. Glutamate is a stimulant of nociceptive neurons. It acts through the activation of receptors present on primary afferents. BTX-A inhibits the inflammatory pain as well as other symptoms by decreasing the release of glutamate peripherally [23]. These effects are observed at a dose below than that used for muscle paralysis [24]. BTX-A inhibits the release of calcitonin gene-related peptide (CGRP), an inflammatory neuropeptide. It reduces the pain response by inhibition of CGRP release from trigeminal nerve [25] and afferent nerve terminals [26]. 

Another mechanism, by which BTX-A leads to reduction in pain, is sensory nerve adaptation. It possibly results from decreased neuroeffector secretions from mast cells, blood vessel endothelium, and sensory nerve tissue [27]. Cholinergic or sympathetic urticaria is a condition characterized by edema, sensory discomfort, erythema, and heat release. The syndrome is considered to involve degranulation of mast cells to release histamine. Histamine and other neuroeffectors possibly lead to sensory disturbances and neurogenic inflammation [28, 29]. Blockage of sympathetically mediated urticaria by BTX-A applied topically over face indicates that a nonneuromuscular mechanism exists which alleviates the symptoms of this condition [19]. 

## 6. Review of the Literature

Botulinum toxin (BTX) has been used in the past for the treatment of regional dystonias associated with pain or sensory disturbance. Botulinum toxin injections to treat spasmodic torticollis showed efficacy in relieving pain far greater than other symptoms of the syndrome [30, 31]. The observations led to further studies to evaluate the role of botulinum toxin in the management of nondystonic pain syndromes, that is, tension headaches and myofacial pain [32]. 

Borodic et al. performed an open-label pilot study to evaluate the efficacy of BTX-A in the treatment of TN. A total of 11 patients suffering from trigeminal neuralgia were treated with BTX-A. Nonresponsiveness to at least 3 drugs was an important inclusion criterion for the study. Multifocal injections were given over the dermatome which is painful. Multiple sites spaced at 10 mm distance were injected to cover the painful anatomic region. Injections were given transcutaneously with depth of injection penetration ranging from 1 to 3 mm. The average dose of botulinum toxin type-A (BTX-A) used was 25–75 units (7.5 U per puncture site). They recommended that anatomic site for injection should be based on the patient's description and anatomical localization of pain on facial region. Brow and upper and lower eyelid injections should be avoided as they can lead to diplopia and ptosis due to diffusion of toxin into the orbit. Patients were evaluated at the 2nd and 6th weeks. The criteria for success were based on 50% reduction in pain intensity and frequency, reduction in the use of medication, and the patient's desire for further injection therapy based on perception of improvement with the first injection. Patients who respond favourably to the treatment are defined responders, and others were named nonresponders. They reported success in 8 patients (out of 11 patients of TN) for a period of 2–4 months. 

The reported complications were transient facial asymmetry during dynamic movements, slight erythema, or edema of skin at injection site. They recommended the use of lower doses initially to avoid facial weakness (criteria to limit the dose of botulinum toxin). They concluded that botulinum toxin type-A (BTX-A) is generally efficient at lower doses to alleviate facial pain. They also stated that their study was a pilot study and there is a need for a well-controlled study design to confirm these results [19].

Türk et al. performed a randomized open-ended study to evaluate the effectiveness of botulinum toxin type-A (BTX-A) in patients with trigeminal neuralgia refractory to other treatment modalities. Eight patients were injected with 100 units of botulinum toxins in the region of zygomatic arch. Based on positive outcomes and lack of any significant adverse effects, they concluded that BTX-A can be utilized in cases of refractory trigeminal neuralgia [24]. 

Piovesan et al. reported successful outcomes with the use of botulinum toxin type-A (BTX-A) in patients of trigeminal neuralgia. The botulinum toxin was injected transcutaneously among the branches of trigeminal nerve. The patients were evaluated using visual analogue scale (VAS) for pain relief on follow-up visits. Out of 13 patients enrolled in the study all patients showed improvement with peak effects reached in 20 days of therapy. Four patients remained pain free, and 9 patients reported partial pain relief with >50% reduction in medication usage. The beneficial effect lasts for approximately 60 days [33]. 

Zúñiga et al. treated twelve patients of idiopathic trigeminal neuralgia which are unresponsive to the medicinal treatment. In this study, the dose of botulinum toxin type-A (BTX-A) ranged between 20 and 50 units. The botulinum toxin is injected subcutaneously in divided doses at various trigger zones along the involved branch of trigeminal nerve. For mandibular division, they recommended additional injection into the masseter muscle. Interestingly, they reported pain relief within few seconds of injection in contrast to other studies which stated that it required some time to develop peak effects. Patients were evaluated at weekly interval for 8 weeks. Visual analogue scale (VAS) was used to evaluate pain prior to injection therapy and after the botulinum toxin injections on follow-up visits. Also, frequency of paroxysmal attacks per day was compared with paroxysms after the injection therapy. The cumulative mean pain score on VAS prior to BTX-A injection was 8.83, and it reduced to 4.08 at the 8th week. Similarly, cumulative number of paroxysmal attacks per 24 hours reduced from 23.42 to 8.67 at the 8th week. The authors reported significant pain relief in 10 patients, while 2 patients did not benefit from BTX-A. The patients remain pain-free for an average period of 60 days. The only disadvantage is transient facial asymmetry due to facial muscle weakness. Although they reported beneficial results, they advocated double-blind studies with larger sample size to validate the utility of BTX-A in the treatment of trigeminal neuralgia [34].

Wu et al. performed a randomized, double-blind, and placebo-controlled study to investigate the safety, tolerability, and efficacy of botulinum toxin type-A (BTX-A) in the management of trigeminal neuralgia. A total of 42 patients were randomly divided into experimental and placebo group (22 in experimental and 20 in placebo group). Out of 42 patients, 40 patients completed the study. The botulinum toxin was administered by either submucosal or intradermal skin injection. A total of 75 units of BTX-A were injected in divided proportions along the area of pain distribution. Similarly, placebo group received equal volume of normal saline. The outcome of the study was to evaluate the degree of pain relief based on visual analogue scale (VAS) for pain. Also, the frequency of pain attacks per day was recorded. Patients with ≥50% reduction in mean pain score at a 12-week period were considered as responders. Based on this criterion, patients who did not show 50% reduction in mean pain score were categorized as nonresponders to the treatment. On statistical evaluation, 68.18% patients in experimental group were responders as compared to 15.00% in the placebo group. The difference was found to be statistically significant. The authors concluded that botulinum toxin type-A may be a safe, novel, and efficient strategy for the treatment of trigeminal neuralgia [35].

Few case reports in the literature reported benefits of the use of botulinum toxin type-A (BTX-A) in the management of trigeminal neuralgia [36, 37].

*Summary*

 Author: Borodic et al.
 Types of study: open-label pilot study.
 Number of patients in the study: *n* = 11
 Inclusion criteria: idiopathic trigeminal neuralgia refractory to at least 3 drugs.
 BTX-A dose: 25–75 U
 Method and site of injection: transcutaneous injection in the area of pain as described and localized by the patient, spaced at a distance of 10 mm. Depth of injection 1–3 mm.
 Criteria for success:
  1. at least 50% reduction in frequency of paroxysm and intensity of pain,
  2. >50% reduction in dose of medication,
  3. patients' desire for further injection because of the appreciation of the beneficial effects of the first injection.
 Results: eight patients with beneficial effects were designated as responders. Beneficial effects last for a period of 2–4 months.
 Adverse effects: transient facial asymmetry during dynamic movements, slight erythema, or edema of skin at injection site.
 Author: Türk et al.
 Types of study: randomized open-ended study.
 Number of patients in the study: *n* = 8
 Inclusion criteria: idiopathic trigeminal neuralgia refractory to treatment.
 BTX-A dose: 100 U
 Method and site of injection: transcutaneous injection in the area of zygomatic arch.
 Results: eight patients show beneficial effects.
 Adverse effects: mild facial asymmetry.
 Author: Piovesan et al.
 Types of study: open-label study.
 Number of patients in the study: *n* = 13
 Inclusion criteria: idiopathic trigeminal neuralgia refractory to treatment.
 BTX-A dose: 25–75 U
 Method and site of injection: transcutaneous injection among the trigeminal nerve branches.
 Criteria for success:
  1. at least 50% reduction in the dose of medication,
  2. reduction in pain intensity as measured by VAS.
 Results: four patients were pain free and 9 patients have >50% reduction in the medication dose. Beneficial effects last for a period of 60 days.
 Adverse effects: transient facial asymmetry.
 Author: Zúñiga et al.
 Types of study: open-label study.
 Number of patients in the study: *n* = 12
 Inclusion criteria: trigeminal neuralgia refractory to medicinal treatment.
 BTX-A dose: 20–50 U
 Method and site of injection: subcutaneously in area of trigger zone. For mandibular involvement additional injections in masseter muscle.
  Criteria for success:
  1. total lack or significant reduction in pain intensity on VAS,
  2. decrease in frequency of paroxysmal attacks of pain per 24 hours.
 Results:
  1. ten patients show significant reduction in pain (VAS). Beneficial effects last for a period of 60 days,
  2. cumulative mean pain score on VAS decreased from 8.83 to 4.08 at week 8,
  3. cumulative number of paroxysm per 24 hr decreased from 23.42/day to 8.67/day.
 Adverse effects: transient facial asymmetry.
 Author: Wu et al.
 Types of study: randomized, double-blind, and placebo-controlled study.
 Number of patients in the study: *n* = 42 (22 in BTX group, 20 in placebo group)
 Inclusion criteria: trigeminal neuralgia.
 BTX-A dose: 75 U
 Method and site of injection: intradermal or submucosal injection in the area of pain distribution.
 Criteria for success:
  1. patients with >50% reduction in pain intensity on VAS,
  2. decrease in frequency of paroxysmal attacks of pain,
  3. patients' overall response to treatment.
 Results: patients with positive outcomes were designated as responders. In BTX-A group, 68.18% patients were responders as compared to 15.00% in placebo group.
 Adverse effects: transient treatment-related adverse effects.

## 7. Discussion

The review of the literature reported success with the use of botulinum toxin type-A (BTX-A) in the management of trigeminal neuralgia refractory to other treatment modalities. But a critical appraisal of the review is essential to establish it as one of the treatment modalities. Although the above-mentioned studies favor the use of BTX-A, few points definitely need to be discussed. 

In the study conducted by Borodic and Acquadro, trigeminal neuralgia patients have characteristic features of inflammatory phenomenon, that is, erythema and edema along with pain [19]. These inflammatory features are not regular features of trigeminal neuralgia [38].

Nurmikko and Cruccu critically reviewed the studies related to positive outcomes of botulinum toxin type-A (BTX-A) in trigeminal neuralgia [19, 24, 33, 37]. In a brief communication, they reported that, the patients involved in these studies may not be true representative of typical case of trigeminal neuralgia. They also reported that there is great confusion regarding the use of botulinum toxin, dose to be injected (2 to 50 units per site), depth of injection (intramuscular and subcutaneous), and onset of effect within hours to weeks after injection and duration of effects (5 weeks to over 6 months). The criterion for successful outcome was not standardized. It was based on variable parameters like frequency of painful attacks and reduction in pain intensity based on VAS. They concluded that, until double-blind randomized controlled trials with typical cases of trigeminal neuralgia have been carried out, there is little advantage of including botulinum toxin in the management of trigeminal neuralgia [39].

Also, the studies conducted by Borodic and Acquadro [19], Piovesan et al. [33], Zúñiga et al. [34], and Wu et al. [35] used visual analogue scale (VAS) to assess reduction in pain intensity. This scale for evaluation was not recommended by the International Association for the Study of Pain. This association has provided some guidelines to measure the outcomes in pain trials [40]. Zakrzewska et al. have suggested specific guidelines to measure the outcomes after the treatment of trigeminal neuralgia [41].

In a brief communication, Zakrzewska et al. critically analyzed the study conducted by Piovesan et al. [33] regarding the efficacy of botulinum toxin type-A (BTX-A) in the management of trigeminal neuralgia. They reported that the treatment group in the concerned study may not be representative of true trigeminal neuralgia sufferers. They concluded that this study lacks scientific merits [42]. Voller et al. performed a randomized double-blind, placebo-controlled study to evaluate the analgesic effect of BTX-A. They concluded that, BTX-A failed to produce any effect on primary or secondary hyperalgesia in several groups [43]. 

## 8. Conclusion

Trigeminal neuralgia is a clinical entity characterized by severe pain. Various conservative medicinal, minor, and major surgical procedures were used in the past with variable success. Unfortunately, few patients failed to respond to these established treatment modalities. Various new treatment alternatives have been tried to provide permanent cure to the patient with minimal morbidity and mortality. Botulinum toxin type-A is one of the recent treatment alternatives. Limited number of case reports and open-ended studies reported favorable outcomes in this regard. But these studies lack scientific merits. Based on the review of the scientific literature, it can be concluded that the scientific literature is insufficient to definitely establish the efficacy of botulinum toxin type-A in the management of trigeminal neuralgia. Further studies with larger sample size are required in this regard.
